# Micropropagation of *Origanum acutidens* (HAND.-MAZZ.) IETSWAART Using Stem Node Explants

**DOI:** 10.1155/2013/276464

**Published:** 2013-07-28

**Authors:** Mehmet Ugur Yildirim

**Affiliations:** Department of Field Crops, Faculty of Agriculture, Ankara University, Diskapi, 06110 Ankara, Turkey

## Abstract

*Origanum acutidens* (HAND.-MAZZ.) IETSWAART is a promising ornamental plant that can be widely used in landscape management. It is endemic to Eastern Anatolian region of Turkey. Tissue culture has not been used to micropropagate it. The study reports stem node explants from one-week-old seedlings of the plant for successful micropropagation. The stem nodes were cultured on MS medium containing 0.6, 1.2, 1.8, and 2.4 mg/L BAP with 0.2 mg/L NAA. Visible effects of culture media on shoot proliferation were recorded. Shoot regeneration rate was maximum on MS medium containing 1.80 mg/L BAP-0.2 mg/L NAA. The micropropagated shoots were rooted on MS medium containing 0.2 mg/L NAA. All microrooted plantlets survived during acclimatisation on peat moss. It was concluded that *O. acutidens* can be successfully micropropagated under *in vitro* conditions.

## 1. Introduction

*Origanum acutidens* (HAND.-MAZZ.) IETSWAART is a perennial herbaceous plant endemic to Eastern Anatolian region of Turkey and has beautiful small light pink to white flowers that open during July-August period each year [1]. They grow on limestone and calcareous rocks and slopes need very little water for their growth and maintenance. Sweet scent from leaves is admired since long times and the intensely aromatic leaves are prized to make their great potential for use in urban landscaping and as ornamental border plant in rock gardens. It is also used as aromatic plants, since ancient times for their preservative and medicinal attributes, as well as to impart flavor to foods [2]. 

There is need to develop methodologies for multiplication and spreading of this plant for the benefit of people. Overexploitation of the plant from natural resources is acting negatively on the populations of the plant and large reserves of the plant that were visible a few years back are no longer visible these days [3]. This suggests that the plant could be used as ornamental in landscaping, pharmaceutical, or food industry after development of protocols for its multiplication through traditional or modern biotechnological methods. Plant tissue culture can act as a possible alternative, which may allow rapid propagation for commercial purpose. In line with this, the study aimed to develop an efficient mass proliferation protocol *O. acutidens* using stem node explants, which has never been reported earlier.

## 2. Materials and Methods

### 2.1. Plant Material and Surface Sterilization

The seeds of *O. acutidens* were collected from the botanical gardens of the Department of Field Crops, Ankara University, Ankara, Turkey during 2012. Soon after collection, the seeds were surface sterilised in 5% NaOCl for sterilization. This was followed by 3 × 5 min rinsing with sterilised bidistilled water. 

### 2.2. Isolation of Explants and Determination of the Best Dose for Sterilisation

The seeds were germinated on 35 mL of MS [4] medium supplemented with 30 g/L sugar and solidified with 6.2 g/L agar (Duchefa) for one week. Once the seeds germinated, they were allowed to grow for one week to obtain miniseedlings. The first stem node from the bottom was used as explant. These stem nodes were cultured on 35 mL MS shoot regeneration medium containing 0.6, 1.2, 1.8, and 2.4 mg/L BAP with 0.2 mg/L NAA supplemented with 30 g/L sucrose. Each culture medium was solidified using 0.62% (w/v) agar (Duchefa) cultured at 24°C in sterile Magenta GA^7^ Vessels under Philips-day light lamps TLD 36 W/54, Hungary with light intensity of 35 *μ*mol m^−2^ s^−1^ under 16 h light photoperiod. 

The micropropagated shoots were rooted on MS medium containing 0.2 mg/L NAA supplemented with 30 g/L sucrose and solidified with 6.2 g/L agar for 28 days in Magenta GA^7^ Vessels under 16 h light photoperiod. 

The pH of shoot and root induction medium was adjusted to 5.7 ± 0.1 with 1 N NaOH or 1 N HCl before autoclaving. All cultures were autoclaved at 121°C, 117.679 kPa for 20 min. 

Once the plants rooted, the agar sticking to the roots was removed under tap water and the plantlets were moved to plastic pots containing peat moss. These plants were acclimatised at 20–22°C in the greenhouse under 16 h light photoperiod and 80% humidity.

### 2.3. Statistical Analysis

The regenerating shoots on each culture medium counted after 45 days to evaluate the regeneration potential of the stem node explants are used in the study. Each experimental treatment had 3 replicates containing 5 explants. Any experimental data taken in percentages were arcsine transformed [5] before statistical analysis. The data related to shoot regeneration was analyzed using one-way ANOVA followed by comparing means using Duncans multiple range test that was performed using statistical software IBM SPSS 20.0 for windows. 

## 3. Results

### 3.1. Effects of Different Concentrations of BAP-NAA on Shoot Regeneration

The mean percentage of shoot regeneration, number of shoots per explant, and mean shoot length varied in each regeneration medium and showed sharp statistical differences (*P* < 0.01) among them on each concentration of BAP-0.2 mg/L NAA (Table 1). The results showed that different concentrations of BAP-0.2 NAA mg/L are effective combinations for the regeneration of shoots from stem node explants. 

The shoot induction started with swelling of explants followed by initiation of shoot buds after one week of culture. These shoot buds gradually developed into fully developed shoots after 45 days of culture. No shoot regeneration was recorded on control (MS medium). Analysis of results showed that shoot regeneration was recorded on all combinations of BAP-NAA with 86.67 to 100% shoot regeneration. Maximum shoot regeneration percentage was recorded on MS medium containing 1.8 mg/L BAP-0.2 mg/L NAA and 2.4 mg/L BAP-0.2 mg/L NAA.

Number of shoots per explant ranged from 2.59 to 9.31 that increased consistently with each increase in the concentration of BAP-0.2 mg/L NAA in MS medium. Each increase in the concentration of BAP-0.2 mg/L NAA had sharp increase in the number of shoots per explant. Minimum and maximum number of 2.59 and 9.31 shoots per explant was recorded on MS medium containing 0.6 mg/L BAP-0.2 mg/L NAA and 2.4 mg/L BAP-0.2 mg/L NAA, respectively.

Shoot length ranged from 0.56 to 2.36 cm that increased consistently from 0.6 mg/L BAP-0.2 mg/L NAA to 1.8 mg/L BAP-0.2 mg/L NAA. Thereafter, with an increase in concentration to 2.4 mg/L BAP-0.2 mg/L NAA, a sharp decline in the shoot length was observed. Minimum and maximum mean shoot length of 0.56 cm and 2.36 cm per explant was recorded on MS medium containing 0.6 mg/L BAP-0.2 mg/L NAA and 1.8 mg/L BAP-0.2 mg/L NAA, respectively.

## 4. Rooting

Well-developed shoots with mean length of 0.56–2.36 cm were rooted on MS medium containing 0.2 mg/L NAA (Table 2). The results showed that initial length of shoots did not affect rooting. Rooting was noted on all explants. However, initial length of shoots affected root length per explant. The number of roots per explant ranged from 1.00 to 2.50 and root length had range of 0.67 to 5.91 cm; such that the maximum number of 2.50 roots per shoot with 5.91 cm long roots was noted on shoots regenerated on MS medium containing 1.8 mg/L BAP-0.2 mg/L NAA. It was followed by 2 roots per shoot with 3.22 cm length noted on shoot regenerated on MS medium containing 2.4 mg/L BAP-0.2 mg/L NAA.

These plantlets were transferred to compost contained in culture vessels and incubated in glass house under ambient conditions of temperature and humidity for acclimatisation. All plants transferred to glass house acclimatised with features of growth and flowered.

## 5. Discussion

There are few reports on mass proliferation of *Origanum*. First study on callus culture of *O. spyleum* was made by Akçam and Yürekli [6]. Tissue culture on *O. bastetanum* [7, 8] and *O. vulgare* [9, 10] has also been carried out previously. However, there is no report on multiplication of the plant through *in vitro* techniques; therefore, propagation technique is of particular importance in *O. acutidens*. 

The present study reports the effect of stem node explants on regeneration for developing an easy and reliable protocol for shoot regeneration explants. 

It is thought that the variants of BAP-NAA may have variable effect on the regeneration and rooting. In line with the hypothesis, the experimental results showed that any concentration of BAP-NAA was suitable for regeneration. It is thought that explants cultured on MS medium contain 0.6–1.8 mg/L BAP-0.2 mg/L NAA. The higher concentration 2.4 mg/L BAP-0.2 mg/L NAA induced negative competition for nutrients due to regeneration of more shoots resulting in reduced length of shoots, whereas the lower concentrations 0.6–1.2 mg/L BAP-0.2 mg/L NAA induced inhibition due to low number and reduced length of shoots. 

The shoots regenerated on BAP-NAA were easily rooted on MS medium in agreement with Socorro et al. [7] and Goleniowski et al. [9]. No abnormality was recorded in the rooted and acclimatized plantlets. This confirms that *in vitro* regenerated *O. acutidens *plantlets could be effectively used for regeneration. Goleniowski et al. [9] reported spontaneous rooting in shoot multiplication medium supplemented with BA (0.28 *μ*M) + NAA (0.53 *μ*M) for *O. vulgare, *whereas Socorro et al. [7] reported on rooting of micropropagated plantlets of *O. bastetanum, *on peat substrate. During the present investigation we obtained rooting in 96% of shoots (an average root length of 5.52 ± 0.2) on medium containing in 0.5 mg/L IBA. 

In conclusion, the results showed that *in vitro* production *O. acutidens* is possible and this plant could be successfully utilized for *in vitro* commercial propagation. It is evident that *in vitro* shoot regeneration and rooting in *O. acutidens *are no longer a problem.

## Figures and Tables

**Table 1:** Effects of various concentrations of BAP-NAA on shoot regeneration from stem node explant of *O. acutiden*.

Regeneration medium		Percentage (%) of shoot regeneration	Number of shoots per explant	Shoot length (cm)
BAP (mg/L)	NAA (mg/L)			
0.6	0.2	86.67^ab^	2.59^d^	0.56^c^
1.2	0.2	86.67^ab^	6.00^c^	0.78^c^
1.8	0.2	100.00^a^	8.45^b^	2.36^a^
2.4	0.2	100.00^a^	9.31^a^	1.81^b^
Control		0.00	0.00	0.00

Values within column followed by different small letters are significantly different at the 0.01 level by Duncan's test. Each value is the mean of 3 replications.

**Table 2:** Effects of various concentrations of regeneration medium on rooting on MS medium containing 0.2 mg/L NAA.

Shoot Regeneration medium		Percentage (%) of rooting	Mean number of roots per explant	Mean shoot length (cm)
BAP (mg/L)	NAA (mg/L)			
0.6	0.2	100.00	1.00^d^	0.67^d^
1.2	0.2	100.00	1.49^c^	2.22^c^
1.8	0.2	100.00	2.50^a^	5.91^a^
2.4	0.2	100.00	2.00^b^	3.22^b^

Values within column followed by different small letters are significantly different at the 0.05 level by Duncan's test. Each value is the mean of 3 replications.
